# Hsp65-Producing *Lactococcus lactis* Prevents Inflammatory Intestinal Disease in Mice by IL-10- and TLR2-Dependent Pathways

**DOI:** 10.3389/fimmu.2017.00030

**Published:** 2017-01-30

**Authors:** Ana Cristina Gomes-Santos, Rafael Pires de Oliveira, Thaís Garcias Moreira, Archimedes Barbosa Castro-Junior, Bernardo Coelho Horta, Luísa Lemos, Leonardo Augusto de Almeida, Rafael Machado Rezende, Denise Carmona Cara, Sérgio Costa Oliveira, Vasco Ariston Carvalho Azevedo, Anderson Miyoshi, Ana Maria Caetano Faria

**Affiliations:** ^1^Departamento de Bioquímica e Imunologia, Universidade Federal de Minas Gerais, Belo Horizonte, Brazil; ^2^Centro Universitário UNA, Belo Horizonte, Brazil; ^3^Instituto Federal do Paraná, Palmas, Brazil; ^4^Departamento de Microbiologia e Imunologia, Universidade Federal de Alfenas, Alfenas, Brazil; ^5^Ann Ronmey Center for Neurologic Diseases, Brigham and Women’s Hospital, Harvard Medical School, Boston, MA, USA; ^6^Departamento de Morfologia, Universidade Federal de Minas Gerais, Belo Horizonte, Brazil; ^7^Departamento de Biologia Geral, Universidade Federal de Minas Gerais, Belo Horizonte, Brazil

**Keywords:** colitis, DSS, IL-10, TLR2, Hsp, *Lactococcus lactis*

## Abstract

Heat shock proteins (Hsps) are highly expressed at all sites of inflammation. As they are ubiquitous and immunodominant antigens, these molecules represent good candidates for the therapeutic use of oral tolerance in autoimmune and chronic inflammatory diseases. Evidences from human and animal studies indicate that inflammatory bowel disease (IBD) results from uncontrolled inflammatory responses to intestinal microbiota. Hsps are immunodominant proteins expressed by several immune cells and by commensal bacteria. Using an IBD mouse model, we showed that oral pretreatment with genetically modified *Lactococcus lactis* that produces and releases *Mycobacterium* Hsp65, completely prevented DSS-induced colitis in C57BL/6 mice. Protection was associated with reduced pro-inflammatory cytokines, such as IFN-γ, IL-6, and TNF-α; increased IL-10 production in colonic tissue; and expansion of CD4^+^Foxp3^+^ and CD4^+^LAP^+^ regulatory T cells in spleen and mesenteric lymph nodes. This effect was dependent on IL-10 and toll-like receptor 2. Thus, this approach may open alternative options for long-term management of IBD.

## Introduction

The intestine is the largest surface of contact between the body and the external environment. It represents a major gateway for potential pathogens and also contacts an extensive and diverse microbiota and dietary antigens toward which tolerance must be induced (1). As such, the gut-associated lymphoid tissue is primarily a tolerogenic environment, and several cell types mediate regulatory activity in the gut mucosa. This includes regulatory T cells (Tregs) subsets such as Th3 cells, which produce TGF-β, and T regulatory 1 cells that produce IL-10 and CD4^+^CD25^+^ Tregs, which are characterized by the expression of the transcription factor *forkhead box p3* (Foxp3) (2, 3). Maintenance of homeostasis is critical to the preservation of health and defects in mucosal tolerance leading to chronic disorders such as inflammatory bowel disease (IBD) (4).

Inflammatory bowel diseases, including Crohn’s disease (CD) and ulcerative colitis (UC), are idiopathic human syndromes marked by unrestrained gastrointestinal inflammation (5, 6) that causes diarrhea, abdominal pain, weight loss, and nausea (7). In Western societies, 1 in 1,000 individuals is affected by IBD, making it an important public health problem. Although the causes for IBD are multiple, it likely involves an inappropriate immune response to the commensal microbiota in a genetically predisposed host (8, 9). Abundant clinical and laboratory research has shown that commensal bacteria harbored within mammalian intestines are the targets of inflammatory responses in IBD patients (10). In addition, recent studies identified a detectable difference between the intestinal microbiota present in individuals with CD and UC when compared to healthy controls. Thus, several evidences suggest that a breakdown in the immune tolerance to the microbiota is central to the development of disease (10–12).

Currently, treatments are mainly focused on remission induction. The drugs usually prescribed include aminosalicylates, corticosteroids, immune modifiers, and antibiotics. Although many patients manage successfully the disease progression with conventional medical therapy, some stay refractory to treatment, most will have recurrent activity of disease, and in some cases, surgery may be required (13). One of the challenges for the development of new therapeutic strategies is the fact that the target antigen involved in the initiation and/or perpetuation of the disease is unknown (14). Since inflamed tissues express high levels of heat shock proteins (Hsps), these antigenic molecules are promising candidates for an immune-based IBD therapy. Hsps are intracellular chaperones that play an important role in preventing unwanted protein aggregation during folding and subunit assembly (15–17). Expression of Hsps can be markedly upregulated by all cells under stress conditions including increased temperature, exposure to pro-inflammatory mediators, and oxidative stress (18, 19). Indeed, Hsp expression is increased in colon of IBD patients and antibodies as well as pathological T cells reactive to self-Hsp60 have been identified in these individuals (20, 21).

Although found to be a target self-antigen in autoimmunity, Hsps are also involved in the body’s homeostasis. Self-Hsp reactive T and B cell clones can be seen as important parts of a network of regulatory cells in the immune system engaged in homeostasis activities, including tissue maintenance and repair as well as the control of inflammation (22–24). Hsp60 has a strong effect in the survival and function of CD4^+^CD25^+^Foxp3^+^ Treg (25). Consistent with this, anti-inflammatory effects of Hsp in autoimmune disease models such as rheumatoid arthritis (26), type I diabetes (27), multiple sclerosis (28), and atherosclerosis (29) have been reported. Recently, our group showed that Hsp65-producing *Lactococcus lactis* (Hsp65-LL) ameliorated experimental autoimmune encephalomyelitis (EAE) in mice, an effect mainly mediated by LAP-expressing Tregs (30).

Since oral tolerance is a potent way of inducing regulatory cells toward specific antigen (31), the idea of using the oral route to trigger tolerance to an antigen involved in autoimmune or inflammatory diseases comes as an important clinical application of the phenomenon (14, 32, 33). Our group has already shown that oral tolerance is more efficiently induced by continuously feeding the antigen. This regimen of feeding, rather than a single dose of antigen administered by gavage, is able to suppress inflammatory responses and autoimmune disease models even in aged mice (34–36). Two major caveats in this protocol of feeding are the difficulties of such procedure and the amount of antigen needed. We used a new strategy to deliver an endotoxin-free form of Hsp65 directly to the gut mucosa in a continuous feeding fashion by a recombinant *L. lactis* strain (37). *L. lactis* is a non-pathogenic, non-invasive, non-colonizing gram-positive bacterium, which was recently described as presenting probiotic properties, and mainly used to produce fermented food (38). *L. lactis* associated with alloantigens is able to induce antigen-specific tolerance to graft-versus-host disease more efficiently than the soluble antigen (39). Consistent with this, genetically modified *L. lactis* to secret other heterologous proteins, such as IL-10 (40), anti-TNF nanobody (41), and *Yersinia* LcrV protein (42), has been successfully applied to IBD experimental models.

In this study, we demonstrate that Hsp65-LL can completely prevent Dextran sodium sulphate (DSS)-induced colitis in mice. Mechanistically, the immunomodulatory effect of Hsp65-LL is associated with induction of CD4^+^Foxp3^+^ and CD4^+^LAP^+^ Tregs, and it requires IL-10 and toll-like receptor 2 (TLR2) pathways.

## Animals and Methods

### Animals

C57BL/6 mice were obtained from Universidade Federal de Minas Gerais (UFMG), Brazil. TLR4^−/−^, TLR2^−/−^, MAL/TIRAP^−/−^, and MyD88^−/−^ (in C57BL/6 background) were kindly provided by Dr. Sergio Costa (UFMG, Belo Horizonte, Brazil), and IL-10^−/−^ (in 129 Sv/Ev background) was provided by Dr. Donna Marie McCafferty (Calgary University, Calgary, AB, Canada). All mice were maintained in a specific pathogen-free facility at UFMG, Brazil. All animal procedures were performed in accordance with the guidelines from Conselho Nacional de Controle de Experimentação Animal (Brazil, http://www.mct.gov.br/index.php/content/view/310553.html) and approved by the University Ethical Committee for Animal Research (Protocol # 114/2010, CEUA-UFMG, Brazil).

### Construction of Hsp65-LL

As described elsewhere (43), a recombinant *L. lactis* strain NCDO2118 able to secrete *Mycobacterium leprae* Hsp65, using a xylose-inducible expression system, was constructed. The constructed vector (pSEC:*hsp65*) directed the expression of Hsp65 to the extracellular medium. *L. lactis* NCDO2118 harboring an empty vector (p*Xyl*T:SEC without hsp65) was used as a negative control in all experiments.

### Bacterial Strains and Growth Conditions

The *L. lactis* NCDO2118 strains were grown in M17 broth (Difco, Detroit, MI, USA) supplemented with 0.5% glucose (GM17) or 1% xylose (XM17; Synth, São Paulo, São Paulo, Brazil) at 30°C, without agitation. When required, chloramphenicol (10 µg/ml; Sigma-Aldrich, St. Louis, MO, USA) was added to the media.

### Conditions of Xylose Induction

On the first day, a single colony of recombinant *L. lactis* harboring an empty vector (p*Xyl*T:SEC) or recombinant *L. lactis* NCDO2118 (p*Xyl*T:SEC:*hsp65*) was grown at 30°C, without agitation, in 5 ml GM17 containing chloramphenicol (Cm, 10 µg/ml). On the second day, the overnight culture was diluted 1:10,000 in 1% xylose fresh M17 (XM17) supplemented with Cm (10 µg/ml) to provide ideal conditions for bacterial growth and *M. leprae hsp65* gene expression. On the third day, when an optical density of 2.0 at 600 nm was reached, which corresponds to 2.5 × 10^8^ CFU/ml, mice treatment was performed.

### *L. lactis* Administration

During 4 days, C57BL/6, IL-10^−/−^, TLR4^−/−^, TLR2^−/−^, MAL/TIRAP^−/−^, MyD88^−/−^ mice were continuously fed either medium (control group) empty-vector-bearing *L. lactis* (CT-LL) or *M. leprae-*Hsp65-LL. Fresh *L. lactis* total culture (bacteria plus supernatant) obtained as previously described was offered to mice daily. Since each mouse drank about 5 ml of culture per day (data not shown) containing 7 µg/ml (44) of *M. leprae* Hsp65, the total dose of bacteria per mouse was estimated to be 5 × 10^9^ CFU and the total daily dose of *M. leprae* Hsp65 was about 35 µg per mouse.

### Oral Treatment with Zymosan

Alternatively, animals received 35 µg of zymosan (Sigma-Aldrich, St. Louis, MO, USA) dissolved in 5 ml fresh empty-vector-bearing *L. lactis* NCDO2118 total culture per day during 4 days as a control for Hsp65-LL.

### Colitis Induction by DSS

Ten days after the last day of oral treatment with Hsp65-producing *L. lactis*, control *L. lactis*, or zymosan, sex- and age-matched animals received 1.5% (w/v) DSS (40 kDa, ICN no. 160110; MP Biomedicals, Santa Ana, CA, USA) in the drinking water, *ad libitum*, for 7 days to induce colonic inflammation (colitis). The amount of DSS and water consumed per animal was monitored; there was no marked difference between experimental groups.

### Macroscopic and Microscopic Assessment of Colitis

Macroscopic score of DSS-induced colitis was derived by separately scoring three major clinical signs, which were weight loss, diarrhea, and rectal bleeding, 7 days after DSS administration as described by Murthy et al. (45). Loss of body weight was calculated as the difference between the initial and actual weight. Diarrhea was showed as mucus/fecal material adherent to anal fur. The presence or absence of diarrhea was confirmed by examination of the colon following completion of the experiment. Mice were sacrificed and the colons were excised. Diarrhea was defined by the absence of fecal pellet formation in the colon and the presence of continuous fluid fecal material in the colon. Rectal bleeding was defined as diarrhea containing visible blood and gross rectal bleeding and scored as described for diarrhea. The macroscopic score was calculated from the score of the clinical signs using the following formula: (weight loss score) + (diarrhea score) + (rectal bleeding score). Samples of colon were fixed in formalin and processed for microscopic analysis. Hematoxylin-eosin (Doles, Panamá, Goias, Brazil)-stained sections were blindly scored based on a semi-quantitative scoring system described previously (46) where the following features were graded: extent of destruction of normal mucosal architecture (0 represents normal; 1, 2, and 3 represent mild, moderate, and extensive damage, respectively); presence and degree of cellular infiltration (0 represents normal; 1, 2, and 3 represent mild, moderate, and transmural infiltration, respectively); extent of muscle thickening (0 represents normal; 1, 2, and 3 represent mild, moderate, and extensive thickening, respectively); presence or absence of crypt abscesses (0 represents absent; 1 represents present); and the presence or absence of goblet cell depletion (0 represents absent; 1 represents present). Scores for each feature were summed up to a maximum possible score of 11.

### Colon Tissue Preparation and Cytokine Assay

The colon was washed with PBS and placed in a buffer solution (1 ml/g) containing Tween-20 0.05% (Sigma-Aldrich, St. Louis, MO, USA), phenylmethylsulfonyl fluoride 0.1 mM (Sigma-Aldrich, St. Louis, MO, USA), benzethonium chloride 0.1 mM (Sigma-Aldrich, St. Louis, MO, USA), EDTA 10 mM (Synth, São Paulo, São Paulo, Brazil), and aprotinin A 20 KIU (Sigma-Aldrich, St. Louis, MO, USA). Then, it was homogenized, centrifuged at 3,000 *g* for 10 min and the supernatants collected for cytokine assay. Plates were coated with purified monoclonal antibodies reactive with cytokines IL-6, TNF-α, TGF-β (active form), IFN-γ, IL-4, IL-17, and IL-10 (BD Bioscience, San Jose, CA, USA), overnight at 4°C. In the following day, wells were washed, supernatants were added, and plates were incubated overnight at 4°C. On the third day, biotinylated monoclonal antibodies against cytokines (BD Bioscience, San Jose, CA, USA) were added and plates were incubated for 2 h at room temperature. Color reaction was developed at room temperature with 100 μl/well of orthophenylenediamine 1 mg/ml (Sigma-Aldrich, St. Louis, MO, USA), 0.04% H_2_O_2_ substrate in sodium citrate buffer. Reaction was interrupted by the addition of 20 μl/well of 2 N H_2_SO_4_. Absorbance was measured at 492 nm by ELISA reader (Bio-Rad, Hercules, CA, USA).

### Measurement of Intestinal IgA

Levels of IgA were determined in the intestinal lavage. Small intestine was rinsed with 10 ml of cold PBS. Intestinal lavages were centrifuged at 12,000 *g* for 20 min at 4°C, and levels of IgA in the supernatants were determined by ELISA. Briefly, 96-well plates were coated with Ig goat anti-mouse unlabeled antibody in coating buffer (pH 9.6) overnight at 4°C. Wells were washed and blocked with 200 µl PBS containing 0.25% casein for 1 h at room temperature. Samples were added to the plates and incubated for 1 h at 37°C. The plates were then washed, peroxidase-streptavidin goat anti-mouse IgA-HRP (Southern Biotechnology, Birmingham, AL, USA) diluted 1:10,000 was added, and the plates were incubated for 1 h at 37°C. Color was developed at room temperature with 100 μl/well of orthophenylenediamine (1 mg/ml; Sigma-Aldrich, St. Louis, MO, USA) and 0.04% H_2_O_2_ substrate in sodium citrate buffer. The reaction was interrupted by the addition of 20 μl/well of 2 N H_2_SO_4_. Absorbance was measured at 492 nm using an ELISA microplate reader (Bio-Rad, Hercules, CA, USA).

### Lamina Propria (LP) Cell Isolation

*Lamina propria* cells were isolated by a modified version of the method described by Davies and Parrot (47). Briefly, the entire length of large intestine was dissected, opened longitudinally, washed with PBS, and cut into small pieces. Tissue fragments were placed in Petri dishes and washed three times in calcium- and magnesium-free HBSS containing 1 mM dl-dithiothreitol (DTT; Sigma-Aldrich, St. Louis, MO, USA) for 30 min. Supernatants were discarded. After that, tissue fragments were incubated with 100 µl/mL of collagenase II (Sigma-Aldrich, St. Louis, MO, USA) for 60 min at 37°C on a shaker. Supernatants were passed through a 70 µm cell strainer (Falcon, Corning, NY, USA) and then resuspended in medium. Cells isolated from LP were then submitted to flow cytometry labeling.

### Flow Cytometry Analysis

Fluorescein isothiocyanate-conjugated mAbs to CD4 (clone RM4-5, IgG2a); phycoerithrin (PE)-conjugated mAbs to CD4 (clone RM4-5, IgG2a); CD45Rb (clone 16A, IgG2a); Foxp3 (clone R16-715, IgG2a); PE-Cy5-conjugated mAbs to CD4 (clone RM4-5, IgG2a); PerCP-Cy5.5 mAbs to CD25 (clone PC61, IgG1); biotin-conjugated mAbs to CD25 (clone 7D4, IgM); streptavidin-Cy5-chrome and streptavidin-allophycocyanin (APC), rat IgG1, IgG2a, IgG2b, and IgM isotype controls were purchased from BD Biosciences (San Jose, CA, USA). The biotin anti-LAP antibody (clone BAF246, IgG) was purchased from R&D Systems (Minneapolis, MN, USA). Surface staining was performed according to standard procedures at a density of 0.5–1 × 10^6^ cells per 25 µl, and volumes were scaled up accordingly. For Foxp3 staining, cells were fixed in Fix/Perm buffer (eBioscience, San Diego, CA, USA) after the surface staining, followed by permeabilization in Perm buffer (eBioscience, San Diego, CA, USA) and staining for PE-anti-Foxp3 according to the manufacturer’s instructions. Samples were acquired in a FACSCan or in a FACSCalibur cytometer (BD Biosciences, San Jose, CA, USA) and analyzed using the FlowJo software (TriStar, San Carlos, CA, USA). At least 30,000 events were acquired for each sample.

### Statistical Analysis

Results were expressed as the mean ± SEM. Normal distribution of samples was confirmed by the Kolmogorov–Smirnov test. Significance of differences among groups was determined by Student’s *t*-test or analysis of variance (Tukey’s post hoc test). Means were considered statistically different when *p* < 0.05.

## Results

### Oral Administration of Hsp65-LL Prevented Colitis in Mice

**Figure 1:**
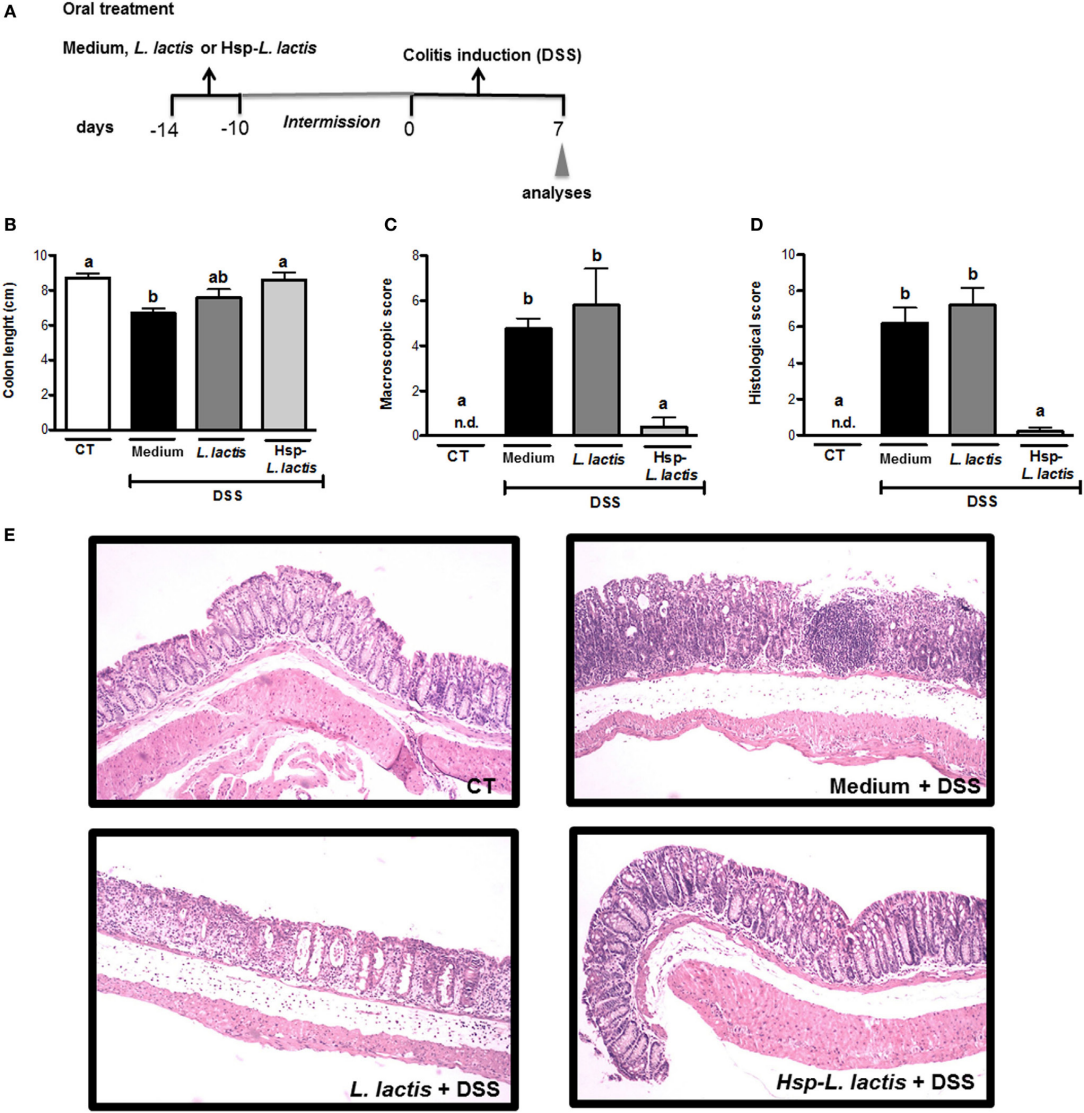
**Effect of Hsp65-producing *L. lactis* (Hsp65-LL) on DSS-induced colitis**. **(A)** Experimental protocol and experimental groups. Groups of five C57BL/6 mice were pretreated or not (naïve) with medium (CT), empty vector-harboring *L. lactis* (CT-LL), or Hsp65-LL for four consecutive days. Ten days later, mice received 1.5% DSS in drinking water for 7 days. **(B)** Colon lengths were measured after 7 days of colitis induction. **(C)** Macroscopic scores including body weight loss, stool consistency, and bleeding were calculated at day 7. **(D,E)** At day 7, colons were collected and dissected for histological analysis by hematoxylin and eosin staining. Histological scores (sum of loss of mucosal architecture, cellular infiltration, muscle thickening, crypt abscess formation, and goblet cell depletion) were ranked blindly. **(E)** Representative distal colon section of naive group (non-treated wild-type mice), CT + DSS, CT-LL + DSS, and Hsp65-LL + DSS. Results are representatives of four independent experiments. Bar graphs are shown as mean ± SEM. Analysis of variance, Tukey’s post hoc test, *p* < 0.05. Distinct letters are used to distinguish groups that are statistically different.

We previously constructed a recombinant *L. lactis* strain able to produce and secrete Hsp65 from *M. leprae*. This *L. lactis*-expressing Hsp65 preparations contained less endotoxin (lipopolysaccharide, LPS) than the limit set by the Food and Drug Administration (34). To verify the efficacy of this new therapeutic concept, C57BL/6 wild-type (WT) mice were continuously fed Hsp65-LL for 4 consecutive days. The total dose of bacteria per mouse was estimated to be 5 × 10^9^ CFU and the total daily dose of *M. leprae* Hsp65 was about 35 µg per mouse. The control groups received either medium (CT) or empty vector-bearing *L. lactis* (CT-LL). After 10 days, mice received 1.5% DSS dissolved in drinking water during 7 days to induce colitis (Figure 1A). We showed that DSS-induced colitis led to colon shortening in mice, which was prevented by the pretreatment with Hsp65-*L. lactis* (Figure 1B). Macroscopically, mice fed Hsp65-*L. lactis* showed similar score (weight loss, diarrhea, and rectal bleeding) to naïve mice (Figure 1C). DSS-induced colitis caused epithelial damage, loss of goblet cells, and crypts, and cellular infiltration starting at day 3. Accordingly, the histological score, devised to allow quantification of histological changes, revealed that pretreatment with Hsp65-*L. lactis* completely prevented the loss of mucosal architecture and inflammatory cell infiltration in mucosa and submucosa layers of the mice (Figures 1D,E).

### Oral Administration of Hsp65-LL Maintained Physiological Levels of Cytokine Production in Colonic Tissue

**Figure 2:**
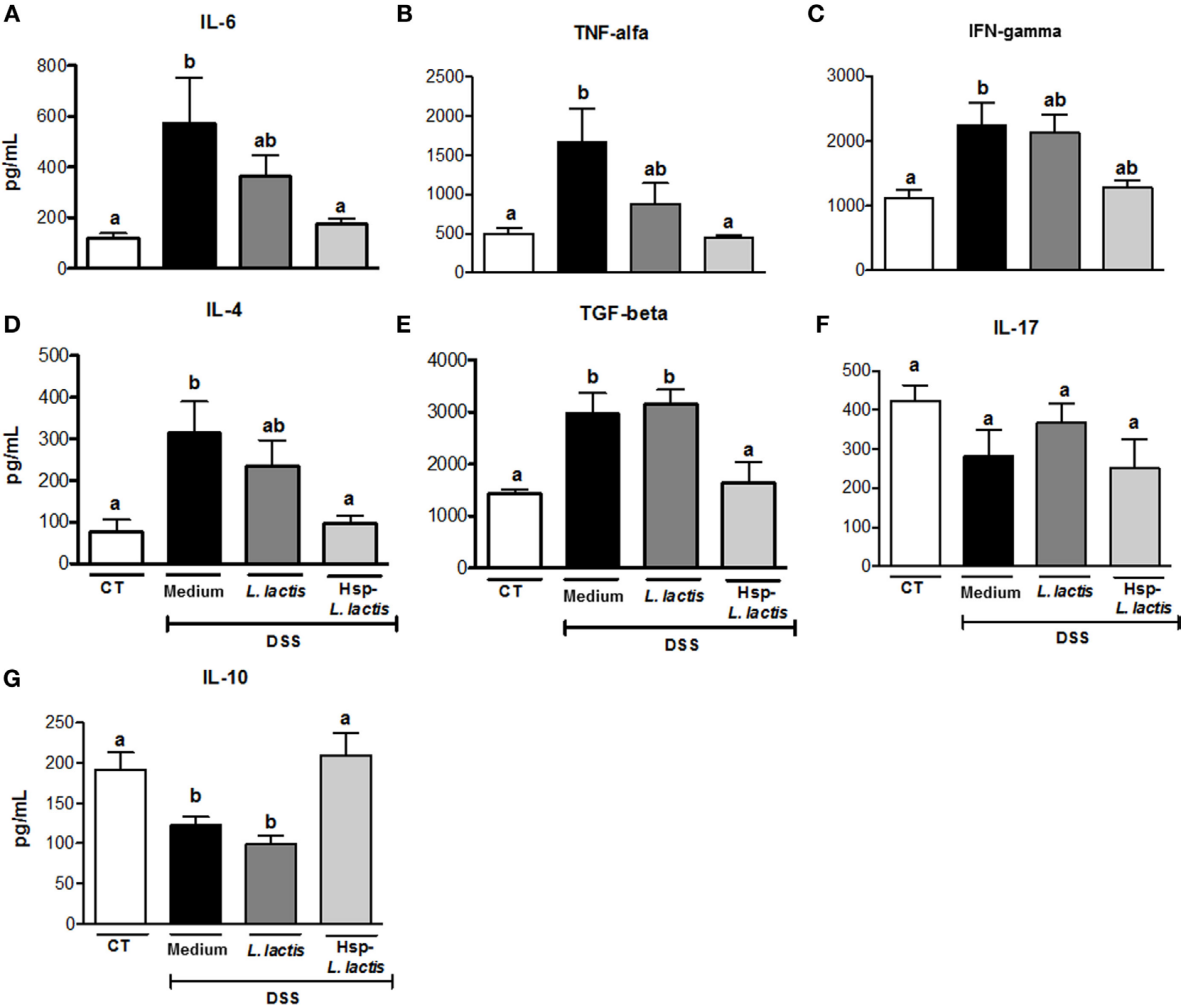
**Effect of Hsp65-producing *L. lactis* (Hsp65-LL) on cytokine production in colonic tissue**. Groups of five C57BL/6 mice were pretreated or not (naïve) with medium (CT), empty vector-harboring *L. lactis* (CT-LL), or Hsp65-LL for four consecutive days. Ten days later, mice were given 1.5% DSS in drinking water for 7 days. Concentrations of cytokines were determined by ELISA. **(A)** IL-6, **(B)** TNF-α, **(C)** IFN-γ, **(D)** IL-4, **(E)** TGF-β, **(F)** IL-17, and **(G)** IL-10. Results are representatives of three independent experiments. Bar graphs are shown as mean ± SEM. Analysis of variance, Tukey’s post hoc test, *p* < 0.05 (*N* = 4). Distinct letters are used to distinguish groups that are statistically different.

We started investigating the mechanisms involved in colitis prevention by Hsp65-LL by assessing whether oral administration of this recombinant bacterium could alter cytokine production in colon tissue. The chosen cytokines are either implicated in the pathogenesis of IBD (IL-6, TNF-α, IL-4, IFN-γ, or IL-17) or have anti-inflammatory activity (IL-10 and TGF-β1). Cytokines were measured in the colonic tissue homogenates after 7 days of DSS administration. We found that levels of IL-6, TNF-α, and IL-4 were increased in the CT group, but they resembled the naïve group in Hsp65-*L. lactis*-treated mice (Figures 2A,B,D). Mice pretreated with empty vector-bearing *L. lactis* showed intermediate levels of these cytokines (Figures 2A,D). IL-17 production did not change in any group when compared with naïve mice, whereas IFN-γ levels were increased by DSS administration, but not reduced by the oral pretreatments (Figures 2C,F). The increase of TGF-β1 levels observed in the CT group was prevented by the pretreatment with Hsp65-*L. lactis* (Figure 2E). However, IL-10, an important cytokine involved in the homeostasis of gut mucosa (48), was kept close to physiological levels (naïve group) after pretreatment with Hsp65-*L. lactis*. Conversely, IL-10 levels found in the CT and CT-LL groups were reduced (Figure 2G). Thus, Hsp65-*L. lactis* oral treatment maintained most of the cytokines measured at physiological levels.

Secretory IgA exerts protective functions in the intestinal mucosa such as improving gut immunological barrier and controlling commensal microbiota (49). Upregulation of its production is a possible mechanism of action for probiotic bacteria. Thus, our next step was to measure IgA in the intestinal lavage of mice. Neither acute colitis nor the oral pretreatment with either empty vector-bearing *L. lactis* or Hsp65-*L. lactis* affected intestinal IgA production (data not shown), suggesting that cytokine maintenance, particularly IL-10, at homeostatic levels rather than secretory IgA underlied the anti-colitogenic functions of oral treatment with Hsp65-*L. lactis*.

### IL-10 Is Required for the Immunomodulatory Effect of Hsp65-LL

**Figure 3:**
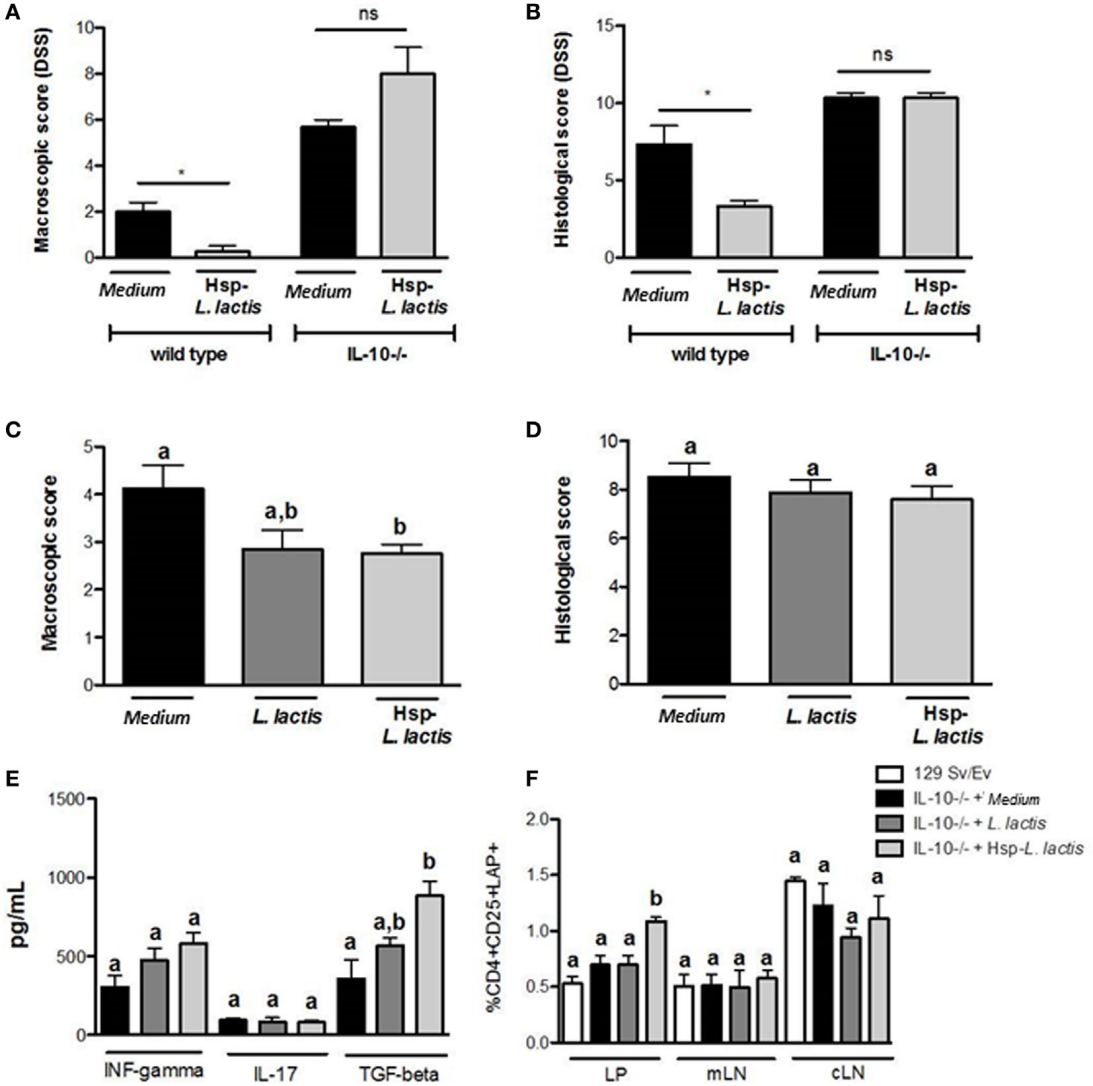
**Anti-colitogenic effect of Hsp65-producing *L. lactis* (Hsp65-LL) is dependent on IL-10**. Groups of 6-week-old IL-10-deficient (IL-10^−/−^) mice or control (129Sv/Ev) mice were pretreated or not (naïve) with medium (CT) or Hsp65-LL for four consecutive days. Ten days later, mice were given 1.5% DSS in drinking water for 7 days. **(A)** Macroscopic score including body weight loss, stool consistency, and bleeding were scored at day 7. **(B)** At day 7, colons were collected and dissected for histological analysis by hematoxylin and eosin staining. Histological scores (sum of loss of mucosal architecture, cellular infiltration, muscle thickening, crypt abscess formation, and goblet cell depletion) were ranked blindly (*N* = 4). **(C,D)** Groups of 6-week-old IL-10-deficient mice were pretreated or not (naïve) with medium (CT), empty vector-harboring *L. lactis* (CT-LL), or Hsp65-LL for four consecutive days. When IL-10^−/−^ mice completed 12 weeks of age, macroscopic and histological scores were ranked. **(E)** Cytokines such as IFN-γ, IL-17, and IL-10 were measured in colon extracts at the end of the experiment. **(F)** Lamina propria (LP) T cells from entire colon, cells isolated from mesenteric lymph nodes (mLNs) or from cecal lymph nodes (cLN) were obtained from IL-10^−/−^ mice. Frequency of CD4^+^CD25^+^LAP^+^ T cells was assessed by flow cytometry and gated in the lymphocyte population (*N* = 8). Results are representatives of two independent experiments. Bar graphs are shown as mean ± SEM. Analysis of variance, Tukey’s post hoc test, *p* < 0.05. Distinct letters are used to distinguish groups that are statistically different. Asterisks are used in panels **(A,B)** to mark statistically significant differences between groups that received medium and Hsp65-LL.

Since Hsp65-LL maintained the physiological levels of IL-10, we investigated whether the prevention of colitis by Hsp65-*L. lactis* was dependent on this anti-inflammatory cytokine. To address this question, 6-week-old IL-10-deficient (IL-10^−/−^) mice and WT mice were orally fed with either medium or Hsp65-LL for four consecutive days. Ten days later, colitis was induced by DSS administration. Different from what we observed in WT mice, macroscopic and histological scores did not change with Hsp65-*L. lactis* oral treatment in IL-10^−/−^ mice (Figures 3A,B), revealing the importance of IL-10 in the protection from DSS-induced colitis.

To confirm the involvement of IL-10 in the anti-colitogenic activity of Hsp65-LL, we tested whether the spontaneous colitis developed in IL-10^−/−^ mice could be ameliorated by the Hsp65-*L. lactis* treatment. In this case, 4-week-old IL-10^−/−^ mice were orally fed Hsp65-*L. lactis* for 4 consecutive days. Macroscopic and histological scores were evaluated after 6 weeks, when IL-10^−/−^ mice show the signals of spontaneous colitis (48, 50). Despite the reduction in the macroscopic score when compared with medium or empty vector-harboring *L. lactis* groups, Hsp65-LL was ineffective in improving the histological score of IL-10^−/−^ mice colitis (Figures 3C,D). Thus, the IL-10 pathway is partially necessary for the Hsp65-LL immunomodulatory effect in the spontaneous colitis suggesting that other mechanisms, such as the production of TGF-β1, may also be involved. Indeed, pretreatment of IL-10^−/−^ mice with Hsp65-*L. lactis* led to an increase in TGF-β1 production in the colon tissue with no changes in IFN-γ or IL-17 (Figure 3E). Consistent with this, TGF-β levels were increased in extracts of colonic *LP*, and the frequency and absolute number of TGF-β1-producing CD4^+^CD25^+^LAP^+^ Tregs were found augmented in the colonic *LP* (Figures 3E,F; Table S1 in Supplementary Material) of IL-10^−/−^ treated with Hsp65*-L. lactis*. Taken together, these data suggest that IL-10 is crucial for the anti-colitogenic function of Hsp65-LL in DSS-induced colitis and at least partially involved in the prevention of spontaneous colitis in IL-10-deficient mice. In the complete absence of IL-10, TGF-β production increased probably as a compensatory mechanism to restore gut homeostasis.

### Prevention of Colitis by Hsp65-LL Is Dependent on TLR2

**Figure 4:**
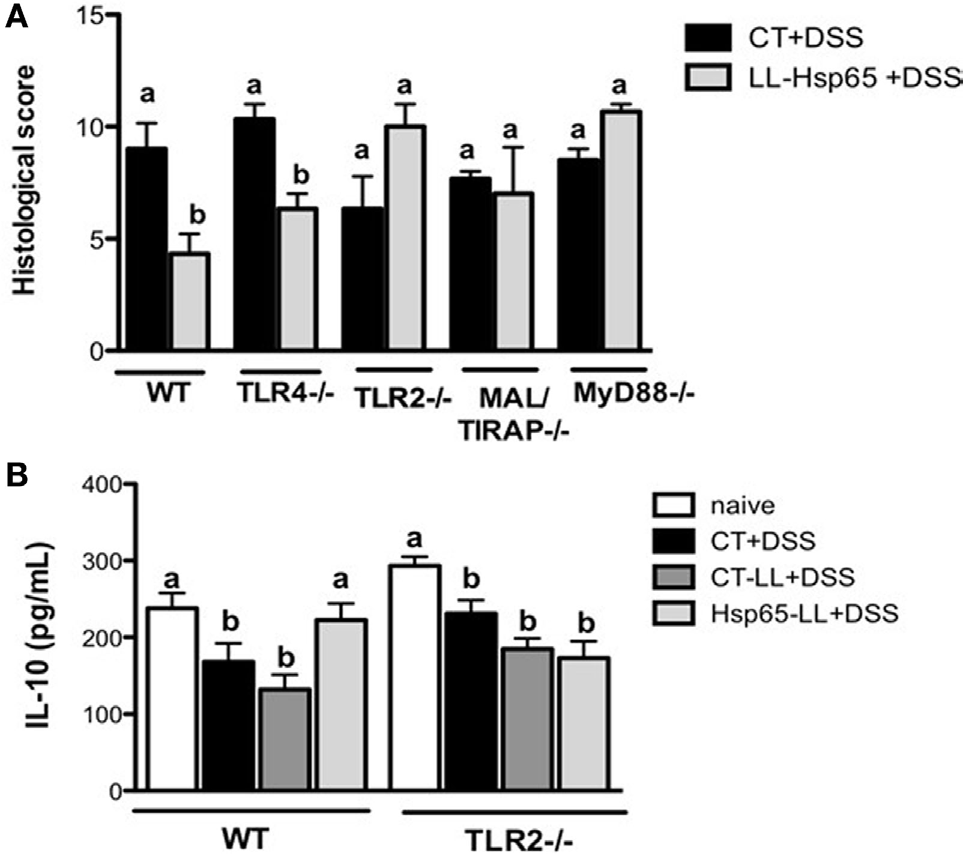
**Toll-like receptor 2 (TLR2) is required for the anti-colitogenic effect of Hsp65-producing *L. lactis* (Hsp65-LL)**. **(A)** Groups of C57BL/6, TLR4^−/−^, and TLR2^−/−^ mice were pretreated or not (naïve) with medium (CT), empty vector-harboring *L. lactis* (CT-LL), or Hsp65-LL for four consecutive days. Ten days later, mice were given 1.5% DSS in drinking water for 7 days. At day 7, colons were collected and dissected for histological analysis by hematoxylin and eosin staining. Histological scores (sum of loss of mucosal architecture, cellular infiltration, muscle thickening, crypt abscess formation, and goblet cell depletion) were ranked blindly. **(B)** IL-10 was measured in colon extracts at the end of the experiment. Results are representative of three independent experiments. Bar graphs are shown as mean ± SEM. Analysis of variance, Tukey’s post hoc test, *p* < 0.05 (*N* = 4). Distinct letters are used to distinguish groups that are statistically different.

Heat shock proteins have the capacity to interact with a variety of immune molecules at the cell surface such as toll-like receptors (TLRs), particularly TLR2 and TLR4 (18, 51). Accordingly, it has been shown that the signaling pathway induced by TLRs is involved in the immunomodulation by immune cells (18, 25, 51). To verify whether the TLR signaling cascade was indeed involved in the immunomodulation of colitis induced by Hsp65-*L. lactis in vivo*, we pretreated TLR2^−/−^, TLR4^−/−^, MAL/TIRAP^−/−^, and MyD88^−/−^ mice with medium, empty-vector-bearing *L. lactis* (data not shown) or Hsp65-*L. lactis*. As shown in Figure 4A, oral administration of Hsp65-*L. lactis* significantly reduced the histological score in WT and TLR4^−/−^ mice, but not in TLR2^−/−^, MAL/TIRAP^−/−^, or MyD88^−/−^ mice, revealing the dependence of these molecules on Hsp65-*L. lactis* anti-colitogenic effects. Consistent with this, IL-10 production was maintained at physiological levels after the pretreatment of WT mice with Hsp65-*L. lactis*, which did not occur in TLR2^−/−^ mice (Figure 4B). To address whether a naturally occurring exogenous TLR2 ligand such as zymozan, a glucan prepared from yeast cell wall, would improve the DSS-induced colitis in a similar fashion than Hsp65, we compared the effect of Hsp65-*L. lactis* with zymozan at the same concentration (35 µg per mouse) dissolved in the CT-LL supernatant. As shown in Figure S1 in Supplementary Material, oral pretreatment with zymozan did not prevent colitis in mice, suggesting TLR2 ligand specificity in the immunomodulation induced by Hsp65-LL.

### Administration of Hsp65-LL Expands Treg

**Figure 5:**
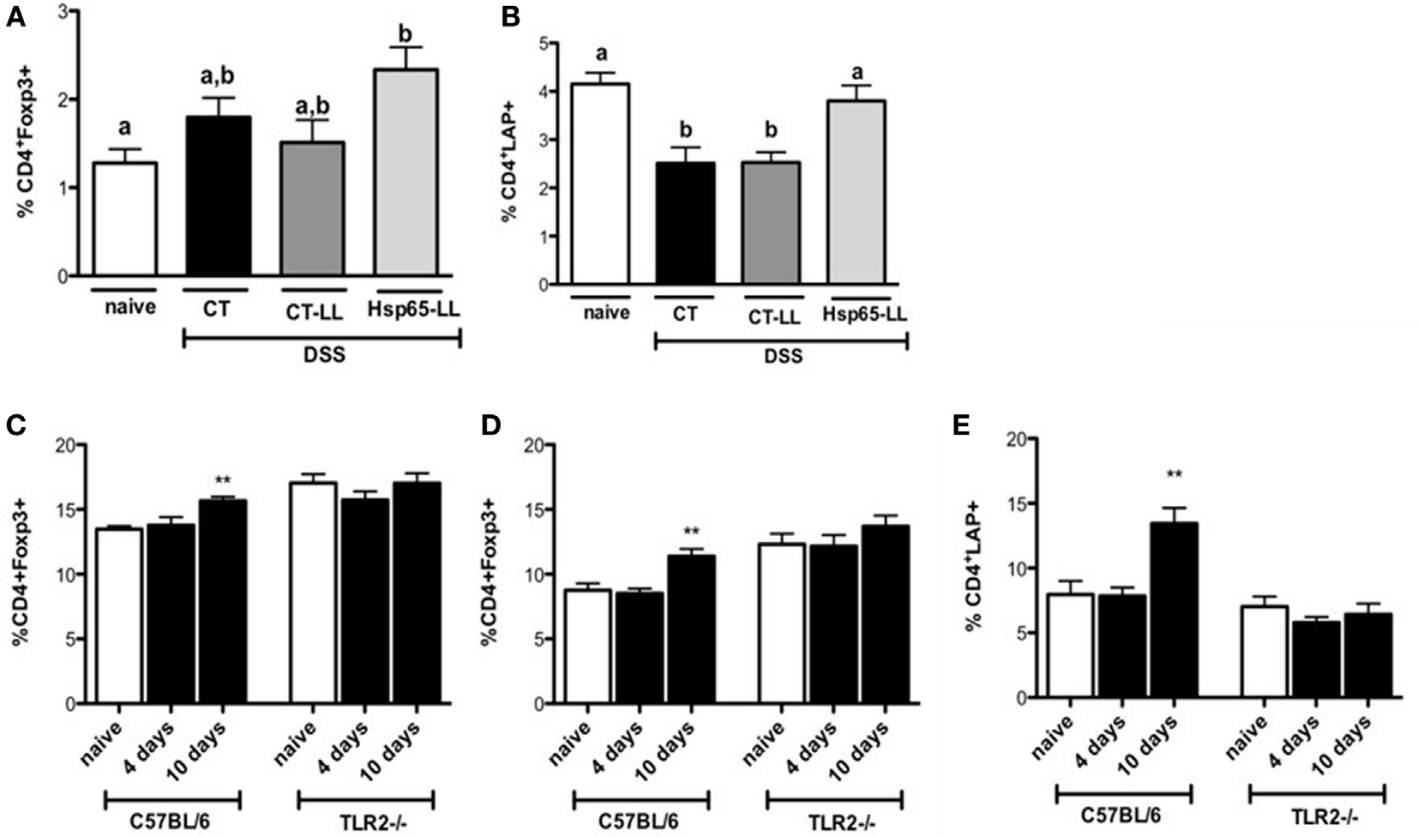
**Hsp65-producing *L. lactis* (Hsp65-LL) induces increased frequencies of regulatory T cells (Tregs) in mice**. C57BL/6 mice were pretreated or not (naïve) with medium (CT), empty vector-harboring *L. lactis* (CT-LL), or Hsp65-LL for 4 days and colitis was induced 10 days later by 1.5% DSS in drinking water. Seventy-two hours after DSS administration, mice were sacrificed and mesenteric lymph nodes (mLNs) removed. Cells were stained with fluorescein isothiocyanate-conjugated (FITC)-anti-CD4, PE-anti-Foxp3, Bio-CD25 and CY5.5-STV **(A)** or with FITC-anti-CD4, PE-anti-CD25, Bio-LAP, and Cy5.5-STV **(B)**. Alternatively, C57BL/6 mice were orally pretreated or not (naïve) with Hsp65-LL during 4 days. Either 4 or 10 days after the last day of oral treatment frequencies of Treg populations were analyzed by flow cytometry. **(C)** Spleen and **(D)** mLN cells were stained with Cy5.5 anti-CD4 and PE anti-Foxp3. **(E)** Spleen cells were stained with Cy5.5 anti-CD4; biotin anti-LAP, and APC streptavidin. CD4^+^Foxp3^+^ and CD4^+^LAP^+^ cells were gated on lymphocyte population (*N* = 4). Results are representative of three independent experiments. Bar graphs are shown as mean ± SEM. Analysis of variance, Tukey’s post hoc test, *p* < 0.05. Distinct letters are used in panels **(A,B)** to distinguish groups that are statistically different. Asterisks are used in panels **(C–E)** to mark statistically significant differences between naïve group and the group that received Hsp65-LL.

Since Hsp65-LL induced an anti-inflammatory cytokine profile in the colon, we next investigated whether Hsp65-LL would affect the frequency of Tregs. Recently, we showed that the pretreatment with Hsp65-LL modulates EAE in mice by inducing CD4^+^Foxp3^+^ and CD4^+^LAP^+^ Tregs (30). In DSS-induced colitis model, the pretreatment of mice with Hsp65-LL increased the CD4^+^Foxp3^+^ cell frequency in the spleen when compared to naïve mice after 3 days of colitis induction (Figure 5A). Moreover, the reduced frequency of CD4^+^LAP^+^ cells observed in both CT and CT-LL groups was restrained by the Hsp65-*L. lactis* pretreatment (Figure 5B). Finally, we verified whether oral pretreatment of mice with Hsp65-*L. lactis* would be sufficient to increase Tregs before the colitis induction and whether TLR2 would be involved in such effect. WT and TLR2^−/−^ mice received the Hsp65-*L. lactis* by the oral route during 4 days, and 4 or 10 days after the last day of oral treatment we analyzed the frequencies and absolute numbers of Treg populations. We observed an increase in CD4^+^Foxp3^+^ (Figures 5C,D) and CD4^+^LAP^+^ (Figure 5E) cell frequencies and numbers (Tables S2 and S3 in Supplementary Material) in spleen and mesenteric lymph nodes (mLNs) of Hsp65-*L. lactis* fed mice 10 days after the oral treatment. However, such Treg increase in WT mice after Hsp65-*L. lactis* pretreatment was not observed in TLR2^−/−^ mice, suggesting the involvement of TLR2 in the Treg expansion after Hsp65-*L. lactis* oral administration.

## Discussion

Heat shock proteins constitute a functional class of highly homologous proteins found in all living organisms and upregulated when cells are under stress (15). Apart from functioning as chaperones, Hsps also present immunoregulatory activities (24, 52). Consistent with this, it has been shown that mucosal administration of Hsp65-induced Tregs in several models of chronic inflammatory conditions such as atherosclerosis (29), rheumatoid arthritis (26), and diabetes (27). Since upregulation of self-Hsps is part of the inflammatory response in many diseases, the induction of cross-reactive T cells against self-Hsp upon mucosal administration of the bacterial Hsp65, becomes an attractive explanation for the development of Tregs in the mucosa. This implies that Hsps may be a therapeutic target for the induction of mucosal tolerance in cases where the auto-antigen is unknown. This is particularly relevant in IBD because the disease is driven by an aberrant immune response to commensals (5).

The precise role of Hsps in IBDs remains unclear. Elevated levels of Heat shock protein 70 (Hsp70) have been found in human biopsies from active UC patients and correlated with disease severity (53). Polymorphisms in the Hsp70-2 gene have been reported as indicators of the clinical course of UC and CD (54). Autoantibodies to Hsp60 and 70 have also been detected in patients with IBD, supporting the concept of immune cross-reactivity between prokaryotic and eukaryotic Hsps as the basis for disease pathogenesis (54). Interestingly, Leung and coworkers (55) demonstrated that in DSS-induced colitis model, luminal bacteria expressed Hsp60 after 3 days of DSS administration, whereas the Hsp60 expression appeared in macrophages at day 6 and in damaged epithelial cells at day 15. Thus, the Hsp60 could be considered as a relevant antigen in IBD and also in DSS-induced colitis.

In this study, we showed that oral administration of Hsp65-LL prevented colitis development in mice and induced CD4^+^Foxp3^+^/CD4^+^LAP^+^ Tregs in a TLR2/IL-10-dependent fashion. Although oral tolerance had been successfully used before in the treatment of experimental IBD (56), no study has yet demonstrated the effects of oral tolerance to Hsps in these diseases.

Recently, we demonstrated that oral administration of Hsp65-LL induced oral tolerance in the model of EAE in mice. Moreover, the effect was associated with increased IL-10 production in mLN and spleen cell cultures and Treg expansion in the same lymphoid organs (30). IL-10 is an important anti-inflammatory cytokine produced by Tregs and a variety of other cell types including epithelia, activated macrophages, dendritic cells (DC), and B1 cells. The importance of this cytokine in shaping mucosal immune responses has been demonstrated by the spontaneous onset of gut inflammation in IL-10-deficient mice (47, 49). Accordingly, we showed that Hsp65-*L. lactis* prevented the reduction of IL-10 in the colonic tissue, which was critical for the immunoregulatory effect induced by the Hsp65-LL in the DSS colitis model. Moreover, despite the improvement in macroscopic score and the increased levels of TGF-β1 and CD4^+^LAP^+^ Tregs found in the colonic tissue of 12-week-old IL-10^−/−^ mice fed Hsp65-*L. lactis*, inflammatory cell infiltration in the colonic tissue was not prevented by such treatment, pointing out the importance of IL-10 in the immunomodulatory mechanisms induced by Hsp65-LL. Of note, in the spontaneous colitis developed by IL-10-deficient mice, it seems that other mechanisms may also play a role since oral administration of Hsp65-LL still had some protective effect. Since TGF- β1 production was upregulated in the colonic mucosa of these treated animals, it is plausible that, in a chronic inflammatory setting happening in the constitutive absence of IL-10, TGF-β1 acted as a compensatory anti-inflammatory mechanism.

Consistent with the important role of IL-10 in our study, Wieten and coworkers demonstrated that Hsp70 immunization of mice 10 days prior to proteoglycan-induced arthritis delayed the disease onset in an IL-10-dependent manner (26). Furthermore, as observed for IL-10, pro-inflammatory cytokines measured in the colon tissue of DSS-induced colitis mice pretreated with Hsp65-*L. lactis* were kept at physiological levels (when compared to non-manipulated naïve mice), suggesting that the main role of Hsp65-*L. lactis* is to maintain homeostasis in the gut mucosa.

As a stress protein and pathogen-associated molecular pattern, Hsps are mainly recognized by pattern-recognition receptors (PRR) present in immune cells, such as TLRs. TLRs comprise a class of transmembrane PRR that play a key role in microbial recognition, induction of antimicrobial genes and control of adaptive immune responses, being important for intestinal homeostasis regulation in both health and disease (57, 58). TLRs are differentially expressed by many distinct cell types throughout the gastrointestinal tract, including intestinal epithelial cells and antigen-presenting cells (58). Moreover, Hsp60 has been shown to be an endogenous ligand for TLR2 and TLR4 (59). Consistent with this, we found that TLR2 expression was critical for the anti-colitogenic activity of Hsp65-LL, since TLR2^−/−^ mice as well as mice deficient for the TLR2 adaptor molecules MyD88 and MAL/TIRAP, which mediate TLR2 signaling pathway (60), showed no improvement of colitis by the pretreatment of Hsp65-*L. lactis*. Thus, the immunomodulation induced by Hsp65-LL is completely dependent on TLR2 and the adaptor molecules involved in its signaling cascade. Hsp70 is also able to inhibit TNF-α, IFN-γ, and MCP-1 produced by bone marrow-derived DC by suppressing C/EBPβ and C/EBPδ transcription factors in a IL-10- and TLR2-dependent fashion (61), indicating that this may represent a conserved and common pathway of immune-modulation by Hsps. Interestingly, the effect of Hsp70 was distinct from the effect of other TLR2 ligands classified as PAMPs such as bacterial peptideoglycan (61). We have also tested another TLR2 ligand, zymosan, on its ability to reproduce the effects of Hsp65. Zymosan is a glucan with repeating glucose units connected by β-1,3-glycosidic linkages found on the surface of yeast (62). Zymosan was administered orally in combination with *L. lactis* at the same maximum concentration found for Hsp65 released by the recombinant strain (35 µg), and no effect on colitis development was observed. This result suggests that, like Hsp70, binding of Hsp65 to TLR2 in cells at the gut mucosa triggered signaling pathways that are distinct from the ones induced by a typical PAMP. Zymosan-induced responses in macrophages include the production of pro-inflammatory cytokines, arachidonate mobilization, protein phosphorylation, and inositol phosphate formation (62). On the other hand, Hsps have been proposed to exert their function as resolution-associated molecular patterns (RAMPs) rather than PAMPs when binding TLRs. RAMPs are shown to counterbalance acute inflammation and restore immune homeostasis by modulating innate cells. After tissue damage, they can modulate acute inflammation by inducing the production of IL-10 (63). In a study comparing the *in vitro* effects of LPS-free *Mycobacterium tuberculosis* Hsp70 and of LPS on DC, Motta and coworkers demonstrated that Hsp70 effects were opposed to the ones triggered by LPS-contaminated Hsp70. In contrast to LPS, Hsp70 did induce neither DC maturation nor TNF-α production by these cells. Instead, it induced IL-10 secretion and inhibited T cell proliferation (64). It is plausible that endogenous ligands such as Hsps bind with distinct affinity to the same receptors or, alternatively, that PAMPs bind to distinct combination of TLRs and other receptors triggering different signaling pathways.

As for the effects of Hsp60, it has been shown that, by binding to TLR2, it leads to a decrease in TNF-α and IFN-γ production and an increase in IL-10 release by T cells. Hsp60 induces a strong effect in the survival and function of CD4^+^CD25^+^Foxp3^+^ Tregs (25) *via* TLR2. In addition, it efficiently drives the differentiation of CD4^+^CD25^−^ T cell clones derived from juvenile idiopathic arthritis patients into CD4^+^CD25^high^ Tregs (65). A similar ability to drive the differentiation of regulatory CD4^+^Foxp3^+^ Tregs and inhibiting inflammatory diseases such as arthritis and colitis has been associated with DC stimulated with Hsp70 (66). These data suggest that Hsps may serve as therapeutic targets for chronic inflammatory diseases.

We demonstrated that oral administration of Hsp65-LL restored the frequency of Tregs in mice subjected to DSS-induced colitis, and it also expanded Tregs in healthy (without colitis) mice. More importantly, these effects were completely abrogated in IL-10^−/−^ and TLR2^−/−^ mice, indicating that the Treg modulation by Hsp65-LL is dependent on IL-10 production and TLR2 activation. Because Hsp65 is highly analogous to the mammalian Hsp60 and *L. lactis* is a safe bacterium to be used in humans, this work supports the idea that Hsp65-LL may constitute an important tool for the treatment of IBDs. On one hand, it may be possible to take advantage of the ability of this protein to induce Tregs for clinical purposes. On the other hand, antigen delivery by a lactic bacterium with probiotic properties in a continuous feeding mode may add an adjuvant effect to the modulatory abilities of Hsp65.

## Author Contributions

AG-S was responsible for planning and executing the experiments as well as writing the manuscript; RO and TM helped planning and executing of the experiments, AC-J, BH, and LL helped executing the experiments; LA helped with the experiments involving TLR signaling; RR helped planning many of the experiments, discussions, and writing the manuscript; SO helped planning the experiments with TLR pathway; VA and AM constructed the recombinant *L. lactis* and helped with planning the experiments; DC helped with histopathological analysis; AMCF designed and supervised the experiments and wrote the manuscript.

## Conflict of Interest Statement

The authors declare that the research was conducted in the absence of any commercial or financial relationships that could be construed as a potential conflict of interest.

## Supplementary Material

Figure S1 **Oral administration of toll-like receptor 2 exogenous ligand does not prevent DSS-induced colitis in mice**. C57BL/6 mice were pretreated or not with medium, Hsp65-producing *L. lactis* (Hsp65-LL), Hsp65-LL supernatant, or zymozan dissolved in *L. lactis* supernatant for 4 days and DSS was induced 10 days later. **(A)** Colon length was measured after 7 days of colitis induction. **(B)** Macroscopic score including body weight loss, stool consistency, and bleeding were scored on day 7. Bar graphs are shown as mean ± SEM. Analysis of variance, Tukey’s post hoc test (*N* = 4). Distinct letters are used in panels **(A,B)** to distinguish groups that are statistically different.
