# Supplementary figures and images for: Hsp65-Producing Lactococcus lactis Prevents Inflammatory Intestinal Disease in Mice by IL-10- and TLR2-Dependent Pathways
Source: Front Immunol. 2017 Jan 30;8:30. doi: 10.3389/fimmu.2017.00030 (PMC5277002; doi:10.3389/fimmu.2017.00030)

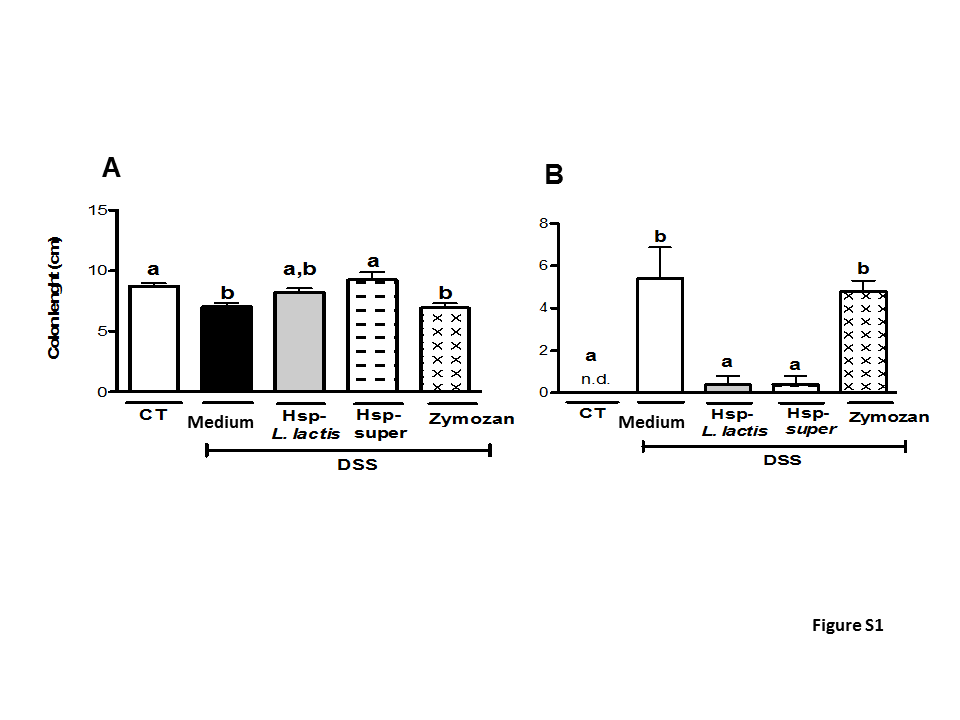

Supplement: Figure S1 — Oral administration of toll-like receptor 2 exogenous ligand does not prevent DSS-induced colitis in mice. C57BL/6 mice were pretreated or not with medium, Hsp65-producing L. lactis (Hsp65-LL), Hsp65-LL supernatant, or zymozan dissolved in L. lactis supernatant for 4 days and DSS was induced 10 days later. (A) Colon length was measured after 7 days of colitis induction. (B) Macroscopic score including body weight loss, stool consistency, and bleeding were scored on day 7. Bar graphs are shown as mean ± SEM. Analysis of variance, Tukey’s post hoc test (N = 4). Distinct letters are used in panels (A,B) to distinguish groups that are statistically different. [file Image_1.TIF]
