# Supplementary material for: Hsp65-Producing Lactococcus lactis Prevents Inflammatory Intestinal Disease in Mice by IL-10- and TLR2-Dependent Pathways
Source: Front Immunol. 2017 Jan 30;8:30. doi: 10.3389/fimmu.2017.00030 (PMC5277002; doi:10.3389/fimmu.2017.00030)
Supplement: Supplementary file 2 [file Table_1.DOCX]

| **Groups** | **LP** | **mLN** | **cLN** |
| --- | --- | --- | --- |
| 129 Sv/Ev | 3.95 X 10^5^± 0.34 **a** | 2.72 X 10^6^± 0.41 **a** | 3.22 X 10^6^± 0.70 **a** |
| IL-10-/- Medium | 4.5 X 10^5^± 0.43 **a** | 3.02 X 10^6^± 0.56 **a** | 3.75 X 10^6^± 0.43 **a** |
| IL-10-/- *L. lactis* | 5.1 X 10^5^± 0.52 **a** | 2.82 X 10^6^± 0.51 **a** | 2.4 X 10^6^± 0.53 **a** |
| IL-10-/- HSP-*L. lactis* | 8.10 X 10^5^ ± 0.9 **b** | 3.56 X 10^6^± 0.67 **a** | 3.25 X 10^6^± 0.83 **a** |

**Table S1** – Number of CD4+CD25+LAP+ T cells in colonic lamina propria (LP), mesenteric lymph nodes (mLN) and cecal lymph node (cLN) of 6-week-old naïve wild type 129 Sv/Ev mice as well as 6-week-old IL-10-/- mice that received either medium, *L.lactis* or Hsp65-producing *L.lactis* for 4 days. Cells were isolated, stained and analysed by flow cytometry 10 days after the last treatment. N=8. Results are representative of 2 independent experiments. Numbers are shown as mean + SEM. ANOVA, post-test Tukey, p<0.05. Distinct letters are used to distinguish groups that are statistically different.
