# Supplementary material for: Hsp65-Producing Lactococcus lactis Prevents Inflammatory Intestinal Disease in Mice by IL-10- and TLR2-Dependent Pathways
Source: Front Immunol. 2017 Jan 30;8:30. doi: 10.3389/fimmu.2017.00030 (PMC5277002; doi:10.3389/fimmu.2017.00030)
Supplement: Supplementary file 3 [file Table_2.DOCX]

| **Groups** | **CD4^+^CD25+Foxp3^+^** | **CD4^+^CD25+LAP^+^** |
| --- | --- | --- |
| Naive | 6.32 X 10^6^± 0.56 **a** | 21.25 X 10^6^± 0.42 **a** |
| CT | 10.34 X 10^6^± 0.43 **a,b** | 11.96 X 10^6^± 0.65 **b** |
| CT-LL | 5.92 X 10^6^± 0.54 **a,b** | 12.98 X 10^6^± 0.51 **b** |
| HSP65-LL | 12.88 X 10^6^ ± 0.76 **a** | 23.13 X 10^6^± 0.43 **a** |

**Table S2** – Number of mesenteric lymph node CD4+CD25+Foxp3+ and CD4+CD25+LAP+ T cells of C57BL/6 mice pre-treated or not (naïve) with medium (CT), empty vector-harboring *L. lactis* (CT-LL) or Hsp65-producing *L. lactis* (Hsp65-LL) for four days. Colitis was induced ten days later by 1.5% DSS in drinking water and mLN cells analysed by flow cytometry 72 hours later. N=4. Results are representative of 3 independent experiments. Numbers are shown as mean + SEM. ANOVA, post-test Tukey, p<0.05. Distinct letters are used to distinguish groups that are statistically different.
