# Supplementary material for: Hsp65-Producing Lactococcus lactis Prevents Inflammatory Intestinal Disease in Mice by IL-10- and TLR2-Dependent Pathways
Source: Front Immunol. 2017 Jan 30;8:30. doi: 10.3389/fimmu.2017.00030 (PMC5277002; doi:10.3389/fimmu.2017.00030)
Supplement: Supplementary file 4 [file Table_3.DOCX]

| **Groups** | **SP CD4^+^FoxP3^+^** | **mLN CD4^+^ FoxP3^+^** | **SP CD4^+^LAP^+^** |
| --- | --- | --- | --- |
| Naive C57BL/6 | 11.7 X 10^6^± 0.34 **a** | 4.1 X 10^6^± 0.41 **a** | 6.96 X 10^6^± 0.34 **a** |
| 4 days C57BL/6 | 10.13 X 10^6^± 0.67 **a** | 4.5X 10^6^± 0.56 **a** | 6.0 X 10^6^± 0.76 **a** |
| 10 days C57BL/6 | 14.40 X 10^6^± 0.52 **b** | 5.9 X 10^6^± 0.51 **a** | 12.02 X 10^6^± 0.51 **b** |
| Naive TLR2-/- | 14.6 X 10^6^± 0.44 **a** | 6.1 X 10^6^± 0.41 **a** | 6.15 X 10^6^± 0.66 **a** |
| 4 days TLR2-/- | 13.12 X 10^6^± 0.22 **a** | 6.6X 10^6^± 0.56 **a** | 6.72 X 10^6^± 0.58 **a** |
| 10 days TLR2-/- | 14.40 X 10^6^± 0.52 **a** | 6.9 X 10^6^± 0.51 **a** | 6.77 X 10^6^± 0.59 **b** |

**Table S3** – Number of CD4+Foxp3+ and CD4+LAP+ Treg cells in spleens (SP) mesenteric lymph nodes (mLN) of C57BL/6 mice orally pre-treated or not (naïve) with Hsp65-producing L. lactis (Hsp65-LL) during 4 days. Either 4 or 10 days after the last day of oral treatment number of Treg cell populations were analyzed by flow cytometry. N=4. Results are representative of 3 independent experiments. Numbers are shown as mean + SEM. ANOVA, post-test Tukey, p<0.05. Distinct letters are used to distinguish groups that are statistically different.
